# Risk Factors for Cerebral Aneurysm Rupture in Mongolia

**DOI:** 10.1007/s00062-021-01051-z

**Published:** 2021-06-30

**Authors:** Matthias Bechstein, Amarjargal Gansukh, Boldbat Regzengombo, Oyun Byambajav, Lukas Meyer, Michael Schönfeld, Helge Kniep, Uta Hanning, Gabriel Broocks, Tserenchunt Gansukh, Jens Fiehler

**Affiliations:** 1 Department of Diagnostic and Interventional Neuroradiology, University Medical Center Hamburg-Eppendorf, Martinistraße 52, 20246 Hamburg, Germany; 2 Mongolian Academy of Sciences, Oyun Onosh Medical Center, Ulaanbaatar, Mongolia; 3 Shastin Central Hospital, Ulaanbaatar, Mongolia

**Keywords:** Subarachnoid hemorrhage, Incidence studies, Risk factors in epidemiology, Underserved populations, Intracranial aneurysm

## Abstract

**Purpose:**

Identification of country-specific demographic, medical, lifestyle, and geoenvironmental risk factors for cerebral aneurysm rupture in the developing Asian country of Mongolia. First-time estimation of the crude national incidence of aneurysmal subarachnoid hemorrhage (aSAH).

**Methods:**

A retrospective analysis of all intracranial digital subtraction angiographies (DSA) acquired in Mongolia during the 2‑year period 2016–2017 (1714 examinations) was performed. During this period, DSA was used as primary diagnostic imaging modality for acute severe neurological symptoms in the sole hospital nationwide dedicated to neurological patients. The catchment area of the hospital included the whole country. Patients with incidental and ruptured aneurysms were reviewed with respect to their medical history and living conditions. The data was used to install a Mongolian aneurysm registry.

**Results:**

The estimated annual crude incidence of cerebral aneurysm rupture was 6.71 for the country of Mongolia and 14.53 per 100,000 persons for the capital region of Ulaanbaatar. Risk factors common in developed countries also applied for the Mongolian population: A medical history of hypertension, smoking or the presence of multiple aneurysms led to a higher relative risk of rupture. In contrast, female gender was not associated with a higher risk in this national cohort. Males pursuing a traditional nomadic living may exhibit a specifically high risk of rupture.

**Conclusion:**

Disease management of over 200 individuals/year with aSAH constitutes a socioeconomic burden in Mongolia. Efforts to raise awareness of the risk factors hypertension and smoking among the Mongolian population are desirable. Measures to improve the nationwide availability of modern neurovascular treatment options are currently under consideration.

## Introduction

The frequency of cerebral aneurysms and risk of rupture with subsequent aneurysmal subarachnoid hemorrhage (aSAH) differ significantly among ethnic groups [1–6]. Nevertheless, knowledge about the incidence of aSAH is limited to high-income and only few middle-income countries. A recent meta-analysis estimated a global yearly crude incidence rate of 6.67 cases per 100,000 persons, with substantial variation across geographical regions [7]. The analysis included only three studies from low and lower middle income countries but a total of 44 studies from upper middle income and high-income countries. This accounts for approximately 6 billion individuals [8], which are missing in global incidence studies on aSAH. Exact knowledge of country-specific disease frequency and risk profiles would facilitate the local implementation of modern neurovascular treatment options. This becomes even more relevant as another recent meta-analysis revealed a global decline in crude SAH incidence by 1.7% annually between 1955 and 2014 but also implied large regional differences particular among Asian countries [9]. Other studies suggested a significant increase in overall hemorrhagic stroke incidence in low and middle-income countries over the last decades, while stroke incidence in high-income countries reduced or levelled in the same period [10–13].

The lower middle income country of Mongolia (year 2017 total population 3.1 million; 3230 US$ per capita gross national income [14]) has so far not been subject of a study on the incidence of cerebral aneurysm rupture leading to SAH. Modern neurovascular treatment options for cerebral aneurysms have just recently been introduced in the capital city of Ulaanbaatar [15].

The Mongolian population is characterized by a variety of unique living conditions [16, 17], possibly impacting the individual risk of aneurysm rupture leading to aSAH. While many live a modern day urban lifestyle in houses or apartments, others permanently reside in urban or rural “Gers” (dwellings that are round wool felt-lined tents), and a minority of the population still pursue an active nomadic living in a harsh geoenvironment. In addition, there are no available scientific data on how the massive air pollution in the capital region of Ulaanbaatar may influence the risk of aSAH [18–21].

The purpose of this study was to: (I) identify the crude annual incidence rates of aneurysm rupture for the country of Mongolia and the capital city of Ulaanbaatar, and (II) reveal country-specific medical, lifestyle and geoenvironmental risk factors.

## Methods

### Data Collection/Mongolian Aneurysm Registry

All patients who underwent an intracranial digital subtraction angiography (DSA) at the emergency department of the Shastin Central Hospital in Ulaanbaatar, Mongolia between January 2016 and December 2017 were retrospectively included. At the time of the study in Mongolia, this hospital served as sole center specialized in neurological emergencies with a nationwide patient catchment area. Furthermore, it was the only institution in the country equipped with an angiography suite (a biplane Philips Allura Xper FD unit, Philips Healthcare, Best, The Netherlands) [22], and a unique local triage setting included the acquisition of a DSA as primary imaging modality for patients with acute neurological symptoms (i.e. severe headache, impaired consciousness) due to lack of other suitable imaging modalities. All angiographies were assessed for presence and number of incidental and ruptured aneurysms by a team of radiologists (all > 10 years experience). To minimize investigator bias, radiologists involved in the image analysis had not performed the DSA. Patient charts were reviewed for demographic factors (age, gender), comorbidities (hypertension, diabetes, history of smoking), geoenvironmental living conditions (urban house, urban ger, rural house, rural ger, nomadic), and field of occupation (agriculture, industrial factory work, mining, outdoor job, office job, other, jobless). In the case of missing data, patients or their dependents were contacted by telephone and the information complemented (with informed consent). The data were then anonymized and therefore consent from patients not contacted by telephone was waived. The local ethics committee approved this protocol.

### Statistical Analysis

Descriptive statistics were applied to estimate epidemiological characteristics. Calculation of the crude incidence rate per 100,000 persons per year was based on population figures retrieved from the World Bank and National Statistics Office of Mongolia for the year of 2017 (total Mongolian population: 3,113,779; population in the capital city of Ulaanbaatar including suburbs: 1,417,396) [14, 23].

We calculated the relative risk (RR) for aneurysm rupture for the following patient parameters (reference parameters in parentheses): age > 50 years (< 50 years), female gender (male gender), hypertension (no hypertension), diabetes (no diabetes), history of smoking (no history of smoking), residence in urban ger, rural house, rural ger (urban house or apartment for all), nomadic living (urban house living), working in agriculture, industrial factory, mining, outdoor, other (office job for all) and with job (jobless). The relative risk ratios, standard error and 95% confidence interval were calculated according to Altman [24].

To test for significance, *p*-values were calculated according to Sheskin [25]. *P*-values ≤ 0.05 were considered significant. Analyses were performed using MedCalc (version 11.5.1.0; Ostend, Belgium).

## Results

In the 2‑year period of this study a total of 694/1714 (40%) of patients who received a DSA had evidence of a cerebral aneurysm. Of these, 418 patients (60%) suffered an aneurysm rupture. The descriptive characteristics of the patient population are displayed in Table 1. Based on population figures for the country of Mongolia at the time of data acquisition (see methods section for exact population data) [14, 23], the following crude incidence rates for aSAH were calculated per 100,000 individuals and year: 6.71 in Mongolia and 14.53 in the capital region of Ulaanbaatar (including suburbs). The national incidence was calculated in consideration of the fact that the observed cohort resembled a national cohort. The hospital included in the study had an extended patient catchment area of the whole country as the only institution nationwide specifically dedicated to the treatment of neurovascular diseases.

**Table 1:** Mongolian patients with cerebral aneurysm

	Ruptured aneurysm (*n* = 418)	Incidental finding (*n* = 276)	*p*-value
*Female*	252 (60)	189 (68)	0.032
*Age*			
< 50 years	182 (44)	127 (46)	0.573
> 50 years	236 (56)	149 (54)	0.573
*Comorbidity/lifestyle*			
Hypertension	270 (65)	156 (56)	0.040
Diabetes	43 (10)	34 (12)	0.484
Smoker	102 (24)	47 (17)	0.022
*Geoenvironment*			
Nomads	12 (3)	4 (1)	0.303
Rural ger	168 (40)	104 (38)	0.558
Rural house	58 (14)	45 (16)	0.446
Urban ger	63 (15)	50 (18)	0.345
Urban house	117 (28)	73 (26)	0.719
*Area of occupation*			
Agriculture	41 (10)	26 (9)	0.969
Outdoor	44 (11)	16 (6)	0.031
Mining	4 (1)	1 (< 1)	0.623
Factory	38 (9)	27 (10)	0.864
Office	62 (15)	43 (16)	0.873
Other/unknown	209 (50)	137 (50)	0.987
Jobless	20 (5)	26 (9)	0.034
*Number of aneurysms*			
Single	300 (72)	219 (79)	0.027
Multiple	118 (28)	57 (21)	0.027
*Reason for imaging*			
Neurological deficit/stroke	395 (95)	10 (4)	< 0.0001
Headaches	13 (3)	210 (76)	< 0.0001
Other	10 (2)	56 (20)	< 0.0001

Data are *n* (%)

Baseline characteristics of all patients diagnosed with at least one cerebral aneurysm between January 2016 and December 2017 in Mongolia

Proportions of patients with arterial hypertension and a history of smoking were more frequent in the ruptured aneurysm group (hypertension: 65% versus 56%, *p* < 0.05; history of smoking: 24% versus 17%, *p* < 0.03). As expected, acute neurological deficits and/or impaired consciousness were more common in individuals with aSAH compared to patients with incidental aneurysm discovered in DSA (95% versus 4%, *p* < 0.0001). Patients with incidental findings of an aneurysm most often received a DSA because of severe headaches. Of all aSAH patients 28% had multiple aneurysms compared to 21% of patients with incidental aneurysm findings (*p* < 0.03). Demographic features, the geoenvironmental living conditions and the occupational background were evenly distributed in both patient groups. Only a minority of the patients who presented to the emergency room pursued an active nomadic living (3% in the ruptured group, 1% in the incidental group, *p* > 0.5), likely being underrepresented due to their limited geographical access to the capital city of this vast country. Permanent stationary living in a traditional Mongolian ger was most common in the rural population; however, not less than 15% (18%) of the urban population included in this study used a ger as their permanent place of residence within the city. Presumably, they resided in the ger districts of the city of Ulaanbaatar, which includes more than 200,000 closely spaced households [16, 17].

Interestingly, a history of smoking was significantly more prevalent among Mongolian males in the overall cohort (46.7% versus 7% among females, *p* < 0.0001), and among males with ruptured and incidental aneurysms (Table 2, *p* < 0.0001 for all subgroups).

**Table 2:** Prevalence of smoking among Mongolians with cerebral aneurysms

History of smoking	Male	Female	*p*-value
All patients	118 (46.7)	31 (7)	< 0.0001
aSAH	80 (47.9)	23 (9.1)	< 0.0001
Incidental finding	38 (44.2)	8 (4.2)	< 0.0001

Data in *n* (%)

**Fig. 1:**
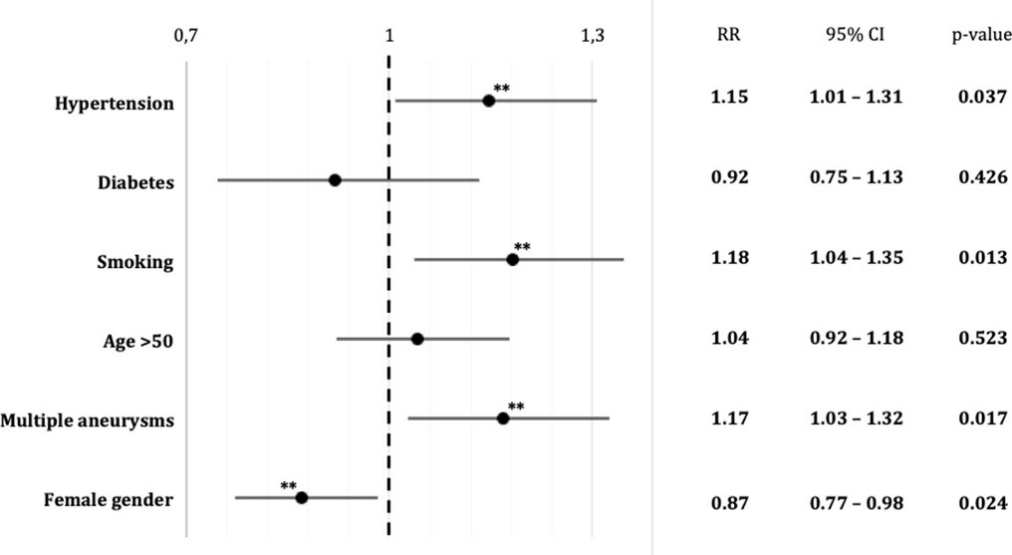
Relative risk (RR) plot of intrinsic risk factors for aSAH. RR of aneurysm rupture by medical preconditions, history of smoking, age (age < 50 years as reference), existence of multiple aneurysms (single aneurysm as reference) and gender (male as reference). RR > 1 indicates an elevated relative risk of rupture. *Double asterisks* indicate a statistically significant increase/reduction

Calculation of relative risk ratios revealed a significant higher risk of aneurysm rupture in Mongolian patients with the comorbidity arterial hypertension (RR 1.15; 95% CI 1.01–1.31; *p* < 0.04) and history of smoking (RR 1.18; 95% CI 1.04–1.35; *p* < 0.02) (Fig. 1). Diabetes as a pre-existing condition was not associated with a significantly altered relative risk of rupture (RR 0.92; 95% CI 0.75–1.13; *p* = 0.43). A reduced risk was observed in female compared to male patients (RR 0.87; 95% CI 0.77–0.98; *p* < 0.03). The occurrence of multiple aneurysms correlated with a significantly elevated relative risk of rupture (RR 1.17; 95% CI 1.03–1.32; *p* < 0.02). While patients above 50 years of age represented the majority in both groups, they did not present with a significantly increased rate of rupture in the hospital.

**Fig. 2:**
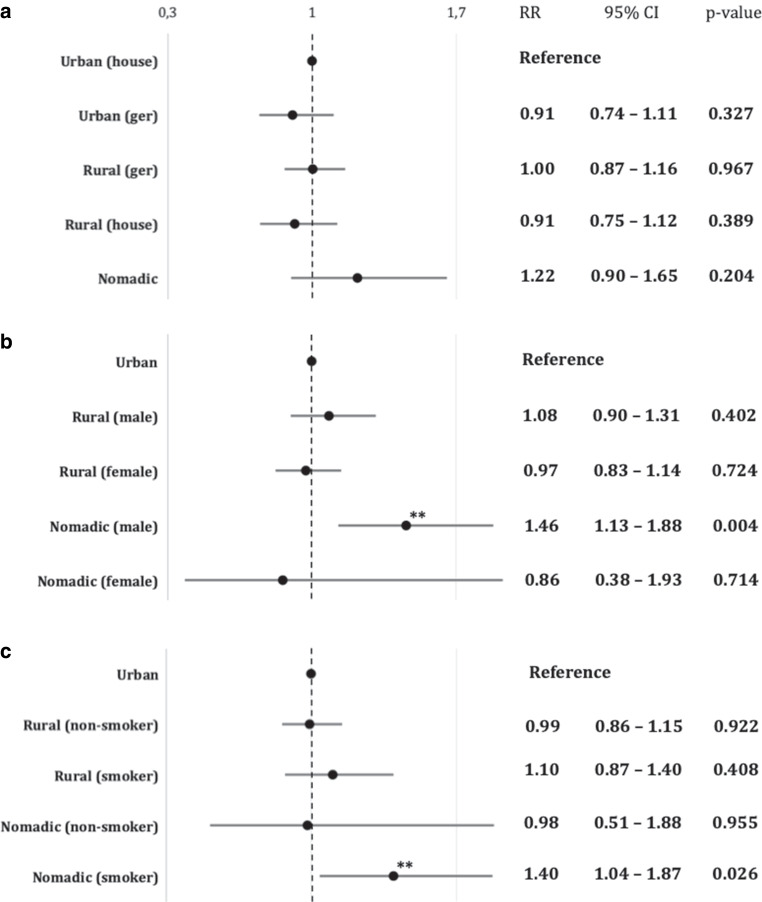
Extrinsic risk factors for aSAH. Relative risk (RR) of aneurysm rupture in different geoenvironmental living conditions in the Mongolian population; Urban living in a house or apartment was used as reference (**a**). **b**, **c** Relative risk for rupture in a rural and nomadic geoenvironment adjusted for gender (**b**) and smoking (**c**). Risk ratios were calculated in reference to urban patients with the same risk factor. RR > 1 indicates an elevated relative risk of rupture. *Double asterisks* indicate a statistically significant increase

Geoenvironmental living conditions had no influence on the risk of rupture in our analysis (Fig. 2a). Nevertheless, after adjustment for the contributing risk factors gender and smoking, nomadic living was significantly associated with more aneurysm ruptures in males as well as in smoking individuals (Fig. 2b, c; RR of 1.46 for nomadic males compared to urban males; 95% CI 1.13–1.88; *p* < 0.005; RR of 1.4 for smokers with nomadic background compared to smokers living in an urban environment, 95% CI 1.04–1.87; *p* < 0.03).

**Fig. 3:**
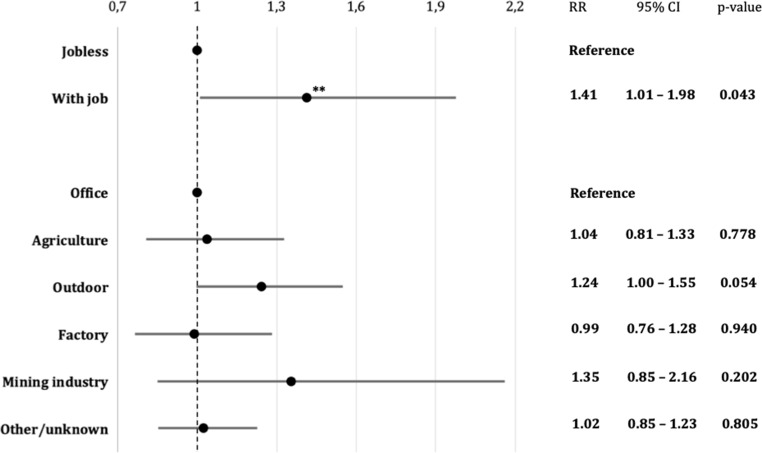
Occupation and risk of aSAH. Relative risk (RR) of aneurysm rupture in Mongolian patients with a job compared to patients without a job. Subanalysis of risk ratios by specific fields of occupation were referenced to patients with an office job. RR > 1 indicates an elevated relative risk of rupture. *Double asterisks* indicate statistical significance

While working patients had relative more ruptures in comparison to jobless patients (RR 1.41; 95% CI 1.01–1.98; *p* < 0.05), subanalysis did not identify any specific area of occupation that correlated significantly with rupture occurrence (Fig. 3). Noteworthy, though, individuals with an outdoor job showed a strong tendency to a higher risk of rupture compared to individuals with an office job (RR 1.24; 95% CI 1.00–1.55; *p* = 0.05).

## Discussion

The estimation of a patient’s risk of aneurysm rupture includes many factors, essentially pre-existing conditions such as hypertension or smoking, but also gender, age, presence of multiple aneurysms, size and location of an aneurysm [2–6, 26, 27]. The widely used and validated PHASES score also includes the population origin of the patient [1]. While North Americans and Europeans other than Finns do not qualify for higher risk scores, patients of Japanese or Finnish origin receive higher risk ratings in this prediction model. Interestingly, the PHASES risk score does not factor in other Asian populations except the Japanese. This is mainly due to a lack of region-specific knowledge about prevalence and incidence rates of aneurysm rupture in Asian populations.

This study is the first to systematically estimate the incidence of cerebral aneurysm rupture in Mongolia. Nevertheless, the annual incidence of 6.71 ruptures per 100,000 persons has to be regarded as a crude estimation, since several infrastructural limitations in this country hinder a true count of all patients with aSAH. A major limitation is the sole use of DSA as primary diagnostic imaging modality for suspected cases of aSAH. Other emergency vascular imaging modalities, e.g. magnetic resonance (MR) or computed tomography (CT) angiography, were not available at the time of the study, neither in the hospital nor, to our knowledge, in any other public or private hospital in the country of Mongolia. In fact, there was a CT scanner installed in the hospital of this study, but due to technical reasons it could not routinely and reliably be used for CT angiography. In addition, as in all hospital-based incidence studies, patients with aSAH who died before admission to a hospital are also not accounted for. Although this is a monocentric study, the patient catchment area of the hospital subject to this analysis included the whole country. Mongolia is vast and covers 1,564,110 km^2^ of area (it ranks 18th globally in terms of geographical size) [28]. Therefore, parts of the population who live in remote provinces with lack of rapid transportation to the central hospital have less chances to be diagnosed with aSAH and are certainly missing in the national incidence calculation. This geographical factor presumably explains the finding of the 2013 update on the global burden of disease, which identified a specifically high mortality of hemorrhagic stroke in Mongolia [11]. Regarding aSAH, a higher than normal grey area of aneurysm ruptures compared to countries with a dense regional hospital network is to be assumed. Nevertheless, the country is also the least densely populated in the world, and urbanization has expanded rapidly with mass migration towards Ulaanbaatar during the last years [17]. Taking this into account, about two thirds of the nation’s population lived within reasonable transportation time to the capital hospital. The calculated aSAH incidence in the city of Ulaanbaatar and its suburbs (14.53 per 100,000) likely reflects a more precise estimation, as access to medical services in the capital is relatively straightforward even in cases of severe aSAH. Nevertheless, there may be a substantial number of patients with undiagnosed aSAH, which were not included in the Mongolian aneurysm registry. The suggested national annual number of 6.71 ruptures per 100,000 persons compares well to 2 recent systematic reviews and meta-analyses, which on a global scale estimated a crude annual aSAH incidence per 100,000 persons/year of 6.67 (95% CI 4.5–9.25) and 7.9 (95% CI 6.9–9.0) [7, 9]. The latter suggested, on a regional level including China and Japan amongst others, a crude incidence of aSAH in Asia of 10.4 per 100,000 person-years. Interestingly, on a country income level, the former meta-analysis estimated an annual incidence in low to middle income countries of only 2.56 (95% CI 1.51–3.86) cases per 100,000, but 8.30 (95% CI 6.72–10.03) cases per 100,000 in high-income countries (for comparison: North America 5.67, 95% CI 3.59–8.21 per 100,000; Europe 7.96, 95% CI 5.78–10.48 cases per 100,000). In that perspective, Mongolia, as a lower-middle-income country, exhibits a higher than average incidence of aneurysm ruptures compared to nations with an equivalent income, and is in line with disease frequency among high-income western populations.

Interestingly, the number of patients with incidental cerebral aneurysms (276 cases, 16% of all patients undergoing DSA in the time period) is noticeably high. Studies from developed countries report prevalences among similar populations with neurological symptoms at 5% with peaks up to 15% in the presence of risk factors [29, 30]. The high number in the present study may be attributable to a selection bias among a patient cohort with severe neurological symptoms, and high prevalence of cardiovascular risk factors. We can certainly not rule out that limited diagnostic modalities in a lower middle income country, in particular inconsistent availability of head CT, no available MRI at the time of study, and higher risk of invasive diagnostics such as lumbar puncture, may have led to missed cases of aSAH with false classification of ruptured aneurysms as incidental aneurysms.

Our study aimed to identify underlying intrinsic (gender, age, medical preconditions) and extrinsic (history of smoking, geoenvironmental living conditions, occupational background) risk factors for aneurysm rupture within the Mongolian population. Since prospective outcome studies are not easy to undertake in a developing country, our risk ratio analysis for these parameters had to rely on descriptive statistics from a retrospective dataset. The calculations of relative risk ratios point to arterial hypertension and smoking as medical and lifestyle factors, which are significantly correlated with higher rupture rates (RR increase to 1.15 and 1.18). This is also well documented among large population studies in other countries [22]. Recent analyses from Ireland and Finland even suggested a decrease of aneurysm rupture incidence with declining numbers of smokers in the last decade [13, 31]. In addition, worldwide SAH decline was paralleled with a decrease of blood pressure in a recent meta-analysis [9]. In modern day Mongolia, high blood pressure constitutes a frequent medical condition with prevalence rates comparable to western societies [32]. Smoking is a very common lifestyle habit in Mongolia and considered to be a major local driver of morbidity and mortality particularly in males [16, 33]. Our findings suggest that, besides tobacco smoke-mediated respiratory and cardiac diseases, more frequent aneurysm ruptures with subsequent aSAH substantially contribute to this condition.

Surprisingly, the results of this study suggest that female gender is linked to a significant decrease in relative risk of rupture among Mongolians. This stands in contrast to aneurysm rupture prediction models, which see females at higher risk of rupture [1]. This effect may be attributable to a high prevalence of smoking in Mongolian males. Another confounder could be a higher male susceptibility to specific adverse geoenvironmental conditions, for instance in the context of a nomadic living or more exposure to outdoor air pollution. Finally, we cannot rule out that the observation of a higher relative risk of rupture in individuals pursuing a nomadic life is an effect of the small sample size of the nomadic population in the registry.

The results of our study are also noteworthy in the context of massive air pollution present in the capital city of Ulaanbaatar [19]. Studies have shown increasing prevalences of respiratory and cardiac diseases and more rates of fetal deaths particularly in the urban Mongolian ger districts, mainly attributable to severely elevated outdoor ambient particulate levels due to use of coal for domestic heating (levels frequently exceed 100 times the WHO recommended safety level) [16, 34]. While we could not identify any relative risk differences for cerebral aneurysm rupture between the two geoenvironmental living conditions urban and rural, individuals with an outdoor job had a strong (RR 1.24; 95% CI 1.00–1.55; *p* = 0.054) but marginally non-significant correlation with higher rupture rates. Those patients might have been more directly exposed to air pollution.

It remains unclear to what extent our findings apply to the Mongolian ethnicity in general. While the nation of Mongolia is relatively small population-wise, the Mongolian ethnicity is widespread across East Asia, mainly in China, but also present in Russia and western societies.

## Conclusion

A crude annual incidence rate of 6.71 aneurysm ruptures per 100,000 persons accounts for > 200 individuals affected each year by this life-threatening disease in Mongolia. Efforts to reduce the widespread medical and lifestyle risk factors hypertension and smoking among the Mongolian society are necessary. Furthermore, the continuous implementation of a countrywide hospital infrastructure with availability of modern neurovascular treatment options is desirable.
